# Collaborative peri-transplant management of volume status, hypertension, and immunosuppression: enhancing kidney transplants for better outcomes

**DOI:** 10.1080/0886022X.2023.2271559

**Published:** 2023-10-27

**Authors:** Amy Perry, Prince Anand Mohan, Kevin Bodker, Mohamed Elshennawy, David J. Taber, Johann Herberth, Karim Soliman

**Affiliations:** a Medical Services, Ralph H. Johnson VA Medical Center, Charleston, SC, USA; b Department of Medicine, Division of Transplant Surgery, Medical University of South Carolina, Lancaster, SC, USA; c Department of Medicine, Division of Nephrology, Medical University of South Carolina, Charleston, SC, USA; d Department of Medicine, Al-Azhar University, Asyut, Egypt; e Department of Surgery, Division of Transplant Surgery, Medical University of South Carolina, Charleston, SC, USA

## Introduction

Accepting marginal kidneys with high kidney donor profile index (KDPI), increases transplantation rates but is associated with substantial risks including delayed or slow graft function (SGF), increased rejection rates and even graft failure. Although there is no universally accepted definition of delayed graft function (DGF), it is commonly characterized by the need for dialysis within the first week after transplantation [1]. Others have described it as the failure of creatinine to decrease by more than 20–30% within the first 24–48 h following surgery, or as little or no urine output after transplant. The latter clinical scenarios are sometimes referred to as SGF when dialysis is not performed [2]. The DGF and SGF rates have significantly increased over the past decades, and many transplant recipients are returning to their dialysis centers until their allografts have recovered or, less frequently, deemed to be primary nonfunctional [3]. DGF/SGF poses additional hazards including worsening hypertension (HTN), volume overload and other complications that are detrimental to graft recovery, psychosocial wellbeing and overall survival [4].

Preventing DGF/SGF and accelerating recovery in graft dysfunction should be approached from a multidisciplinary perspective to ensure the best patient outcomes possible [4]. Our goal is to review the key early pre- and post-transplant factors of blood pressure control, volume management and immunosuppression that influence graft recovery and that are potentially influenceable by collaborative approaches to.

## Pre-transplant volume status

Uncontrolled HTN and fluid overload are well-known major risk factors for all-cause and cardiovascular mortality in the dialysis population [5]. Cardiovascular disease (CVD) affects more than 50% of individuals undergoing dialysis, and the risk of death from CVD events is reported to be 20-times higher compared to the general population [6]. As per the guidelines established by the Kidney Disease: Improving Global Outcomes (KDIGO), it is suggested that hypertensive chronic kidney disease subjects and kidney transplant patients be treated with the objective of achieving a systolic blood pressure (SBP) target below 120 and 130 mmHg respectively, as tolerated, utilizing a standardized office blood pressure measurement method. Targeting euvolemia through daily evaluations is a complex task. Physical exam findings, such as measurement of jugular venous pressure (JVP), S3, hepatojugular reflex have adequate specificity but lack the sensitivity to be used alone [7]. Central venous pressure measurement is commonly used in the inpatient setting but is wrought with error [8]. Natriuretic peptides, such as beta-natriuretic peptide, is often used in heart failure assessment [9,10]. While prognostically helpful in ESKD, its current role in volume assessment in this patient population is debated [11]. The use of novel diagnostic techniques, such as the tumor marker protein CA125, which is primarily used to monitor the progression and treatment response of ovarian cancer, is now emerging as a prognostically valuable tool as well as a way to measure hypervolemia and improve cardiac and renal outcomes. Increased CA125 values may signal fluid retention or abdominal cavity inflammation, necessitating further investigation into the patient’s fluid status [12]. These tests are currently not part of clinical guideline recommendations, and trials are primarily in heart failure making direct extrapolation to the ESKD population difficult. Venous Excess Ultrasonography (VExUS) is an effective volume management method, particularly for patients who are critically ill. The inferior vena cava (IVC) and hepatic veins are examined to evaluate the presence of venous congestion and extra volume, which can help direct fluid removal tactics. By evaluating the IVC and hepatic veins, VExUS can guide clinicians in making decisions regarding fluid removal or diuretic therapy [13]. Combining CA125 and VExUS assessments enables a more thorough approach to volume management because CA125 can help identify non-cardiac causes of fluid retention and VExUS offers real-time information on venous congestion, which enables clinicians to better target treatment for patients with volume-related problems [13]. Point-of-care ultrasonography (POCUS) is becoming increasingly valued in evaluation of volume status in the inpatient setting with the advent and validation of VExUS in 2020 [14]. Given its inherent technical difficulties particularly in the evaluation of intrarenal veins, VexUS adoption may be difficult for those with limited familiarity with POCUS. It should also be noted that the prognostic value of POCUS in predicting renal outcomes have come into question as of recently [15]. As an alternative, the technically less difficult evaluation of JVP by ultrasound (uJVP) was shown in multiple studies to have promising test characteristics for predicting right atrial pressure (right sided filling pressure) [16,17].

Volume overload in the immediate preoperative period can contribute to DGF/SGF. By always maintaining proper EDW, community nephrologists are crucial in contributing to the success of transplants, particularly in marginal kidney recipients. Wait-listed patients should be routinely reminded of the value of following volume control techniques (medication adherence, fluid and diet restrictions, dialysis treatment goals), as they may be called in for a DDKTx at any time. The value of preemptive transplantation before dialysis initiation has been recognized. Since volume overload might jeopardize this benefit, the collaboration between community nephrologists and transplant teams is important because it may be preferable to initiate dialysis prior to transplantation to achieve optimal volume status, particularly if a living donor is available. Hypervolemia should be presumed when the patient exhibits signs of edema, orthopnea or HTN, particularly if they have these at their EDW. EDW should be regularly challenged for potential transplant recipient with uncontrolled BP or edema. As echocardiograms are routinely performed as part of the transplant evaluation, patients with elevated right heart pressures (RVSP > 35) should also be further challenged to achieve euvolemia. Patients with vascular congestions, interstitial edema, or small effusions on chest x-ray also warrant EDW reduction.

## Immediate post-transplant volume and blood pressure considerations

Patients in the immediate post-operative period are a heterogenous population rendering protocolized fluid management susceptible to pitfalls. Intravascular volume depletion can expose patients to acute kidney injury (AKI) secondary to pre-renal physiology or even acute tubular injury. States of excess volume can cause clinical signs and symptoms of congestive nephropathy, congestive heart failure or even precipitation of acute cardiac events. A feedback loop of vascular congestion, graft dysfunction with poor urine output is established. The belief that post-transplant patients should be adequately hydrated to reduce the risk of ischemia-reperfusion can be detrimental in such cases. Volume expansion should be titrated based on individualized assessment of available diagnostic modalities as previously mentioned. A low threshold for early dialysis post-operatively may be an important modality to correct hypervolemia in a timely manner.

In this context, perioperative blood pressures are often elevated far above a patient’s usual baseline because of multiple etiologies. Home antihypertensives are typically held pre-operatively. Abrupt discontinuation of adrenergic blockers can cause rebound HTN. The introduction of high dose steroids and early initiation of a calcineurin inhibitors (CNIs) in the setting of preoperative hypervolemia further contribute to this problem. A major challenge in controlling blood pressure can be the selection of antihypertensives on re-introduction. Use of renin angiotensin aldosterone system (RAAS) blocking agents are generally not utilized in the immediate post-transplant period. Thiazide and loop diuretics have reduced efficacy in DGF/SGF with reduced urine output and can further exacerbate pre-renal AKI. Non-dihydropyridine calcium channel blockers may cause CNI toxicity by inhibiting the cytochrome-p450 system. Overall, a cooperation between community nephrologists and transplant teams is paramount to ensure that medication selection, dosing and changes are continuously and appropriately adjusted as the clinical status indicates. Kidney Disease Improving Global Outcomes (KDIGO) guidelines recommend targeting BP < 140/90 mmHg for patients without proteinuria and <125/75 mmHg for patients with proteinuria. We generally target BP <130/80 mmHg in most patients when the proteinuric status is unknown.

## Immunosuppressive medication considerations

CNIs are nephrotoxic with a narrow therapeutic range [18]. The CNI blood level is affected by many factors such as hypoalbuminemia and hemoconcentration that result in potentially significant drug level variations in DGF/SGF patients particularly in the inter-dialytic periods. The extent of organ exposure to the medication as well as the potential free drug portion remains a mystery leaving the cumulative hazardous effects including nephrotoxicity and neurotoxicity of these drugs uncertain. It is advisable to conduct regular monitoring of whole-blood 12- and 24-h trough concentrations in patients receiving CNIs. Patients on tacrolimus treatment, with the respective timeframes applicable to immediate-release and extended-release preparations, it is recommended to maintain a therapeutic range of 8–10 ng/mL for the first month following transplantation, and 5–8 ng/mL for the subsequent months. The goal trough levels for cyclosporin ranges from 200 to 300 ng/mL over the initial three months following transplantation and 50–150 ng/mL is applicable for the succeeding months. Acute CNI nephrotoxicity includes isometric tubular vacuolization, arteriolopathy, thrombotic microangiopathy, afferent arteriolar vasoconstriction, decreased renal blood flow, glomerular sclerosis, tubular ischemia, dysfunction, and atrophy, interstitial fibrosis and electrolyte disturbances [18]. In addition to the volume management related challenges, body reserve for additional volume can be reduced by extremity amputation. On this background, careful post-discharge CNI serum level monitoring and management is crucial to minimize risk of cardiac events, AKI and delayed allograft recovery. The transplant team should clearly communicate what their goal is for therapeutic drug monitoring, taking into consideration factors such as time since transplant, presence of Donor Specific Antibodies (DSA), age of recipient and risk for adverse effects.

## Proposed stepwise approach for patient management in the peri-transplant time

Table 1 illustrates a proposed strategy for managing volume status, HTN, and immunosuppressive medications in the immediate pre- and post-transplant phase. At our center, each transplant nephrologist is assigned to a specific region, establishing regular communication with the respective community nephrologists. This facilitates seamless cooperations and ensures effective patient care throughout the transplant process. Ensuring that the dialysis staff are aware of wait-listed patients will also aid in optimization.

**Table 1.** A proposed management Strategical scheme for CKD/ESKD patients pre- and post-transplantation.

Pre-transplant: Community Nephrologist to optimize volume status and blood pressure in preemptive and ESKD patients utilizing traditional and innovative techniques. Foster a collaborative approach, coordination between transplant nephrologists and community nephrologists is essential.
Post-transplant: To ensure continuity of care, assign the patient a dedicated transplant nephrologist. Continue the communication between community and transplant nephrologists. Ongoing meticulous volume assessment via various mentioned techniques at the dialysis and transplant centers. Obtain a 24-h urine creatinine once non-oliguric (>400 cc/24 h). Low threshold for obtaining a kidney biopsy 1-2 weeks post-transplant, earlier as clinically indicated. Consider CNI minimization, postpone CNI initiation or CNI avoidance protocols. Low threshold for decremental dialysis for DGF patients.

CNI: calcineurin inhibitor; DGF: delayed graft function; ESKD: end-stage kidney disease

In DGF/SGF patients, the mindset of decremental dialysis post-transplant with a low threshold to wean off dialysis should always be in effect. Post-transplant patients with DGF/SGF may benefit from once or twice weekly dialysis versus a standard thrice weekly schedule. Less frequent dialysis may help control factors such as volume and hyperkalemia while still allowing the kidney ample time to function independently. Once urinary output exceeds 1 L for oliguric patients, or within 2 weeks post-transplant for non-oliguric patients, it is advisable to conduct a 24-h urine creatinine clearance test. Consideration should be given for more than weekly assessment of weight, edema, and blood pressure. Unlike ESKD patients, DGF/SGF patients should not be prescribed a specific EDW without considering the patient’s overall clinical condition. Our primary objective is to prevent intradialytic hypovolemia while also avoiding hypervolemia. Staff should closely montior blood pressure during dialysis and reduce volume removal as hypotension is approached. We suggest making this reduction as SBP approaches 100 mmHg rather than waiting for it be lower than 100 mmHg. This cautious approach seeks to strike a balance in fluid management to ensure optimal outcomes for DGF/SGF patients and is best achieved through constant communication between the community nephrologists and the transplant team.

In the setting of suboptimal graft function, immediate-release CNI is suggested over extended-release CNI due to their difference in half-lives. Long-acting CNI formulations present challenges in achieving and maintaining therapeutic drug levels within a narrow range, as well as promptly addressing any potential toxic levels. It is imperative for the patient, dialysis staff and community nephrologist to be aware which formulation the patient is taking to ensure appropriate timing of labs if troughs are being drawn in the dialysis clinic.

It is important to note that laws regulating dialysis vary across the US and Europe. Although the US dialysis regulations require nephrologists to evaluate ESKD patients once a week, care of DGF/SGF patients necessitates more frequent clinical assessments. This should include volume status, blood pressure control, and potential signs of improved allograft function. The concept of ‘monthly’ dialysis labs is also not appropriate when caring for this patient group and will need more careful coordination with the transplant team. We recommend a minimum of weekly lab work to monitor for changes in serum creatinine and consideration of decremental dialysis when appropriate. Allograft biopsy should be considered only after careful direct discussion with the transplant nephrologist.

The collaborative approach outlined in Table 1 focusing on volume, HTN and immunosuppression together with a personalized and regionally focused assignment of transplant nephrologists can play an important part in efforts to optimize patient outcomes after renal transplantation.
